# The influence of initial symptoms on phenotypes in spinocerebellar ataxia type 3

**DOI:** 10.1002/mgg3.719

**Published:** 2019-05-23

**Authors:** Hao‐Ling Xu, Qiu‐Ni Su, Xian‐Jin Shang, Arif Sikandar, Min‐Ting Lin, Ning Wang, Hong Lin, Shi‐Rui Gan

**Affiliations:** ^1^ Department of Neurology and Institute of Neurology First Affiliated Hospital, Fujian Medical University Fuzhou China; ^2^ Department of Laboratory Medicine The 1st Affiliated Hospital of Xiamen University Xiamen China; ^3^ Department of Neurology Yijishan Hospital of Wannan Medical College Wuhu China

**Keywords:** initial symptoms, neurodegeneration, phenotypes, spinocerebellar ataxia type 3

## Abstract

**Background:**

Spinocerebellar ataxia type 3 (SCA3) is a rare, inherited form of ataxia that leads to progressive neurodegeneration. The initial symptoms could affect clinical phenotypes in neurodegenerative diseases, such as Parkinson's disease and amyotrophic lateral sclerosis. However, the contribution of initial symptoms to the phenotypes of SCA3 has been scarcely investigated.

**Methods:**

In the present study, 143 SCA3 patients from China were recruited and divided into two groups of gait‐onset and non‐gait‐onset. For determining the influences of initial symptoms on age at onset (AAO), the severity and progression of ataxia, and the possible factors affecting the initial symptoms, multivariable linear regression, and multivariate logistic regression were performed.

**Results:**

We found that the frequency of gait‐onset was 87.41%, and the frequency of non‐gait‐onset was 12.59% (diplopia: 7.69%, dysarthria: 4.20%, dystonia: 0.70%). Compared to the non‐gait‐onset group, the gait‐onset group had significantly more severe ataxia (*p* = 0.046), while the initial symptoms had no effect on AAO (*p* = 0.109) and progression of ataxia (*p* = 0.265). We failed to find the existence of any factors affecting initial symptoms.

**Conclusion:**

These findings collectively suggested that initial symptoms influenced phenotypes in SCA3 and that neurodegeneration in different parts of brain may induce different disease severity in SCA3.

## Abstract 2

To investigate the contribution of initial symptoms to the phenotypes of spinocerebellar ataxia type 3 (SCA3), 143 SCA3 patients from China were recruited and divided into two groups of gait‐onset and non‐gait‐onset. We found that compared to the group of non‐gait‐onset, the group of gait‐onset had significantly more severe ataxia. Our finding suggested that initial symptoms influenced phenotypes in SCA3 and that neurodegeneration in different parts of brain may induce different severity in SCA3.

## 1 INTRODUCTION

Spinocerebellar ataxia type 3 (SCA3), also known as Machado–Joseph disease, is the most common subtype of spinocerebellar ataxias (SCAs) and one of the neurodegenerative diseases. SCA3 is caused by a pathological CAG repeat expansion located in exon 10 of *ATXN3 *(OMIM: 607047) on the site of chromosome 14q32.1, which results in an elongated glutamine tract in the ataxin‐3 protein (Durr, 2010; Kawaguchi et al., 1994). Alleles with repeat expansions ranging from 12 to 44 CAG repeats are considered normal, whereas pathogenic expansions occur with more than 50 repeats (Costa Mdo & Paulson, 2012; Gan, Ni, Dong, Wang, & Wu, 2015). SCA3 has presented with various clinical manifestations, such as gait instability, limb incoordination, dysarthria, dysphagia, dysmetria, nystagmus, diplopia, and peripheral neuropathy (Bettencourt & Lima, 2011; Durr, 2010). Though the majority of patients manifests an ataxic gait as the first symptom, the initial symptoms may also include vision abnormalities, loss of limb dexterity, speech disturbances, and peripheral neuropathy (Bettencourt & Lima, 2011; Globas et al., 2008; Luo et al., 2017; Riess, Rub, Pastore, Bauer, & Schols, 2008).

The initial symptoms may have prognostic value in other neurodegenerative diseases, such as Parkinson's disease (PD) and amyotrophic lateral sclerosis (ALS). Compared to postural instability and gait difficulty subtypes, the tremor onset subtype of PD has an earlier age at onset (AAO), slower motor progression, and less cognitive impairment (Aarsland, Andersen, Larsen, Lolk, & Kragh‐Sorensen, 2003; Jankovic & Kapadia, 2001; Jankovic et al., 1990; Thenganatt & Jankovic, 2014; Williams‐Gray, Foltynie, Brayne, Robbins, & Barker, 2007). And the initial symptoms could have an influence on disease progression, functional decline, and survival in ALS (Czaplinski, Yen, & Appel, 2006; Czaplinski, Yen, Simpson, & Appel, 2006; Watanabe et al., 2015; Zoccolella et al., 2008). Nevertheless, the contribution of initial symptoms to the phenotypes of SCA3 has not been thoroughly investigated, except for one study by Luo et al in which it was suggested that initial symptoms in American SCA3 patients do not influence AAO and disease progression (Luo et al., 2017). In addition, the frequency of initial symptoms could be affected by AAO in ALS (Atsuta et al., 2009; Yokoi et al., 2016), however, such factors affecting initial symptoms have never been investigated systematically before.

In the present study, 143 molecularly confirmed SCA3 patients from China were recruited to investigate whether the initial symptoms could affect the AAO, severity, and progression of ataxia, and to determine the factors affecting the initial symptoms.

## 2 SUBJECTS AND METHODS

### 2.1 Ethical compliance

The study was approved by the Ethics Committee of the First Affiliated Hospital of Fujian Medical University. Written informed consents were signed by all participants.

### 2.2 Study subjects

A total of 143 molecular‐confirmed SCA3 patients of Chinese Han nationality were recruited for the present study. The inclusion criteria were (a) the presence of ataxia, (b) clear recall of initial symptoms, (c) willingness of participation, and (d) age of 14 years and older. Exclusion criteria were (a) exclusion of SCA3 by previous genetic tests, (b) homozygotes, and (c) concomitant disorder(s) that affect the International Cooperative Ataxia Rating Scale (ICARS) and other ataxia measurements used in this study.

### 2.3 Genotype and phenotype analyses

Each patient was asked to provide a peripheral blood sample, and the genomic DNA was extracted from blood samples using a QIAamp DNA Blood MiniKit (Qiagen, Hilden, Germany). Polymerase chain reaction and further Sanger sequencing were performed to determine the number of CAG repeats in the *ATXN3* gene, as previously reported (Gan et al., 2015, 2010).

Each patient was interviewed face‐to‐face by ataxia specialists to obtain all information needed for the present study. The initial symptoms were defined as the onset symptoms that were recalled by the patient or close relatives/care providers at the first sign of their disease. The AAO was defined as the age when the patient or close relatives/care providers first noticed any symptoms. The disease duration was the time span between AAO and the age at first visit. The severity of the disease was evaluated by ICARS, and ranged from 0 (absence of ataxia) to 100 (most severe ataxia). Four segments of postural and stance disorders, limb ataxia, dysarthria, and oculomotor disorders composed a complete set of the scale (Trouillas et al., 1997). The progression of ataxia was assessed using the following calculation: the ICARS scores divided by duration (in years).

### 2.4 Statistical analyses

According to the reported initial symptoms, each subject was categorized into either the gait‐onset group or the non‐gait‐onset group. For analyses of basic demographics between the two groups, Chi‐squared tests were used to compare the gender distribution. Two‐independent samples *t* test and Mann–Whitney *U* test were used, respectively, for normal and non‐normal distributed variables, which were confirmed by Kolmogorov–Smirnov testing.

For determining the influence of initial symptoms on AAO, the severity and progression of ataxia, multivariate linear regressions were performed, respectively. In the analysis of the influence of initial symptoms on AAO, AAO was the dependent variable, and initial symptoms (binary) along with gender (binary), and the length of normal and expanded CAG repeats were independent variables. To analyze the influence of initial symptoms on the severity of ataxia, the ICARS score was the dependent variable, and initial symptoms (binary) along with gender (binary), AAO, disease duration, and the length of normal and expanded CAG repeats were independent variables. To analyze the influence of initial symptoms on the progression of ataxia, the progression value was the dependent variable, and initial symptoms (binary) along with gender (binary), AAO, disease duration, and the length of normal and expanded CAG repeats were independent variables. For determining the possible affected factor in relation to initial symptoms, multivariate logistic regression analysis was performed. The initial symptoms (binary) were the dependent variables, and the AAO, gender (binary), the length of normal and expanded CAG repeats were independent variables.

All of the statistical analyses were performed using the R Project (version 3.2.2, 2015 The R Foundation for Statistical Computing). The results were considered statistically significant at *p* < 0.05.

## 3 RESULTS

The demographic features of the SCA3 participants are presented in Table 1. The frequency of gait‐onset in our subjects was 87.41%, while the frequency of non‐gait‐onset was 12.59% (diplopia: 7.69%, dysarthria: 4.20%, dystonia: 0.70%). The gender (*p* = 0.626), AAO (*p* = 0.509), disease duration (*p* = 0.375), length of normal CAG repeats (*p* = 0.518), and expanded CAG repeats (*p* = 0.562) were similar between two groups of gait‐onset and non‐gait‐onset. While compared to the non‐gait‐onset group, the ICARS score in the gait‐onset group was significantly increased (gait‐onset: 34.30 ± 20.43 vs. non‐gait‐onset: 20.97 ± 14.93, *p* = 0.009). The disease progression was prone to be faster in the gait‐onset group than in the non‐gait‐onset group, though there was no significant difference between the two groups (gait‐onset: 4.86 ± 3.68 vs. non‐gait‐onset: 3.41 ± 2.15, *p* = 0.060).

**Table 1:** The demographic features of SCA3 participants

	Variable distribution	Gait‐onset	Non‐gait‐onset	*p*‐Value
Sample size, *N* (%)	/	125(87.41%)	18(12.59%)	NA
Age of first visit, Year	Normal	44.95 ± 12.36	41.44 ± 12.37	0.262^c^
Gender (M/F)		68/57	8/10	0.626^a^
Disease duration, Year	Skewed	9.06 ± 5.89 Median = 8.00	7.52 ± 5.14 Median = 7.00	0.375^b^
Age at onset, Year	Normal	35.88 ± 11.34	34.00 ± 11.05	0.509^c^
Length of expanded CAG repeats, *N*	Skewed	74.73 ± 3.73 Median = 75.00	74.22 ± 3.95 Median = 73.00	0.562^b^
Length of normal CAG repeats, *N*	Skewed	19.58 ± 6.69 Median = 14.00	21.28 ± 7.07 Median = 23.00	0.518^b^
ICARS score	Skewed	34.30 ± 20.43 Median = 30.00	20.97 ± 14.93 Median = 19.50	**0.009** b
Progression of ataxia	Skewed	4.86 ± 3.68 Median = 4.00	3.41 ± 2.15 Median = 3.14	0.060^b^

Values represent mean ± standard deviation or number, and for variables with non‐normal distribution, the median is reported as well.

Bold value showed statistical significance.

Abbreviations: F, female; ICARS, International Cooperative Ataxia Rating Scale; M, male; *N*, number; SCA3, Spinocerebellar ataxia type 3.

a Chi‐square test.

b Mann–Whitney *U* test.

c Two‐independent samples *t* test.

To further examine the possible influence of initial symptoms on AAO, severity, and progression of ataxia (Table 2), we performed multivariate linear regression. After adjusting for AAO, gender, disease duration, and length of normal and expanded CAG repeats, we found that the severity of ataxia in the gait‐onset group was significantly more severe (*p* = 0.046). Nevertheless, there were no significant differences in AAO (*p* = 0.109) and progression of ataxia (*p* = 0.265) between the two groups of gait‐onset and non‐gait‐onset.

**Table 2:** The influences of initial symptoms on AAO, severity, and progression of ataxia

	Coefficient estimate	Standard error	*p*‐Value
The influence of initial symptoms on AAO			
Initial symptom^a^	3.217	1.994	0.109
Gender^b^	2.150	1.321	0.106
Expanded CAG repeats	−2.215	0.179	**0.000**
Normal CAG repeats	−0.002	0.100	0.980
The influence of initial symptoms on ataxia severity			
Initial symptom^a^	7.132	3.549	**0.046**
AAO	0.745	0.151	**0.000**
Gender^b^	−0.461	2.363	0.846
Expanded CAG repeats	1.804	0.457	**0.000**
Normal CAG repeats	−0.087	0.179	0.630
Disease duration	2.395	0.207	**0.000**
The influence of initial symptoms on ataxia progression^c^			
Initial symptom^a^	1.282	0.888	0.265
AAO	0.088	0.376	**0.020**
Gender^b^	−0.659	0.588	0.333
Expanded CAG repeats	0.111	0.115	0.105
Normal CAG repeats	0.072	0.441	0.265

Bold values showed statistical significance.

Abbreviations: AAO, age at onset; ICARS, International Cooperative Ataxia Rating Scale.

a Gait‐onset versus non‐gait‐onset.

b Female versus male.

c Ataxia progression: the ICARS scores divided by disease duration (in years).

We also performed multivariate logistic regression to investigate the possible factors that affect initial symptoms. However, the factors of AAO (*p* = 0.136), gender (*p* = 0.403), and length of normal (*p* = 0.290) and expanded (*p* = 0.114) CAG repeats were found to have no influence on initial symptoms.

## 4 DISCUSSION

In the present study, we found that nearly ninety percent of Chinese SCA3 patients presented slowly progressive gait ataxia as the earliest symptom. Additionally, diplopia was the most common symptom for the non‐gait‐onset group, which was consistent with the studies of American (Luo et al., 2017) and European (Globas et al., 2008) SCA3 patients. This result may indicate earlier disease pathology in the visual/oculomotor centers of the brainstem. Interestingly, there was one patient in our cohort who presented with blepharospasm and oromandibular dystonia recalled that the special dystonia occured 5 years earlier than gait ataxia. Since dystonia was also a common non‐ataxia symptom in SCA3 patients (Kuo et al., 2017; Moro et al., 2014), the initial symptom of this patient was defined as dystonia.

To investigate whether there were risk factors for the initial symptoms, Luo et al. (2017) and Globas et al. (2008) compared the numbers of CAG repeats between the groups of gait‐onset and non‐gait‐onset and found no significant difference in CAG repeat numbers. The present study further expanded the possible factors of AAO, gender, and length of CAG repeat of normal and expanded allele, and investigated whether these possible factors are related to initial symptoms by performing multivariate logistic regression. However, we also failed to find any positive factors that could affect the initial symptoms of SCA3. These results collectively suggested that trans‐acting factors associated with the patients, such as environmental factors, may have effects on the initial symptoms.

Interestingly, in contrast to the study that was based on the analysis of SCA3 patients from American populations (Luo et al., 2017), we found that compared to the non‐gait‐onset group, the gait‐onset group had significantly more severe ataxia. The difference between these two studies may be due to the ethnic and environmental disparity between two SCA3 populations. Our findings suggest that initial symptoms have a marked impact on disease severity in Chinese SCA3 patients. The prognostic value of initial symptoms may also be seen in other neurodegenerative diseases, such as PD and ALS. The tremor onset subtype of PD usually presents with earlier AAO, slower motor progression, and less cognitive impairment than postural instability and gait difficulty subtypes (Aarsland et al., 2003; Jankovic & Kapadia, 2001; Jankovic et al., 1990; Thenganatt & Jankovic, 2014; Williams‐Gray et al., 2007). As well, the disease progression, functional decline, and survival could also be affected by initial symptoms in ALS (Czaplinski, Yen, & Appel, 2006; Czaplinski, Yen, Simpson, et al., 2006; Watanabe et al., 2015; Zoccolella et al., 2008). Neuropathologic studies have revealed that severe and widespread neuronal loss in SCA3 patients' brain, especially in the cerebral cortex, basal ganglia, thalamus, midbrain, pons, medulla oblongata, and cerebellum (Koeppen, 2005; Seidel et al., 2012). The various initial symptoms are suggestive of the damage of different neuropathological substrates at initial stage of SCA3. And the prognostic value of initial symptoms in SCA3 and other common neurodegenerative diseases indicated that initial neurodegeneration in different parts of brain may lead to variously different manifestations in neurodegenerative diseases.

Nevertheless, the present study has some limitations. First, the classification of initial symptoms requires some clarification due to the limitations of study subjects. There were four initial symptoms in our subjects (gait instability, diplopia, dysarthria, dystonia). However, because of the low frequency of dysarthria and dystonia, we had to categorize the initial symptoms into gait‐onset and non‐gait‐onset robustly. Second, the progression assessed in this study requires fine‐tuning. Since we only had data for baseline visits of our subjects, we divided ICARS score by the disease duration to get the disease progression. Therefore, we needed follow‐up visits to get more precise interactions between initial symptoms and disease progression.

Overall, we recruited 143 SCA3 patients from China and found that the majority of SCA3 patients presented with gait instability as the initial symptom and the gait‐onset patients had more severe ataxia compared to non‐gait‐onset patients, suggesting the influence of initial symptoms on phenotypes in SCA3 and different patterns of neurodegeneration at initial stage may induce differential disease severity in neurodegenerative diseases.

## CONFLICT OF INTEREST

All authors report no conflicts of interest.
